# Optimization of Treatment Geometry to Reduce Normal Brain Dose in Radiosurgery of Multiple Brain Metastases with Single–Isocenter Volumetric Modulated Arc Therapy

**DOI:** 10.1038/srep34511

**Published:** 2016-09-30

**Authors:** Qixue Wu, Karen Chin Snyder, Chang Liu, Yimei Huang, Bo Zhao, Indrin J. Chetty, Ning Wen

**Affiliations:** 1 Department of Radiation Oncology, Henry Ford Health System, MI, USA

## Abstract

Treatment of patients with multiple brain metastases using a single-isocenter volumetric modulated arc therapy (VMAT) has been shown to decrease treatment time with the tradeoff of larger low dose to the normal brain tissue. We have developed an efficient Projection Summing Optimization Algorithm to optimize the treatment geometry in order to reduce dose to normal brain tissue for radiosurgery of multiple metastases with single-isocenter VMAT. The algorithm: (a) measures coordinates of outer boundary points of each lesion to be treated using the Eclipse Scripting Application Programming Interface, (b) determines the rotations of couch, collimator, and gantry using three matrices about the cardinal axes, (c) projects the outer boundary points of the lesion on to Beam Eye View projection plane, (d) optimizes couch and collimator angles by selecting the least total unblocked area for each specific treatment arc, and (e) generates a treatment plan with the optimized angles. The results showed significant reduction in the mean dose and low dose volume to normal brain, while maintaining the similar treatment plan qualities on the thirteen patients treated previously. The algorithm has the flexibility with regard to the beam arrangements and can be integrated in the treatment planning system for clinical application directly.

Surgery, chemotherapy, whole brain radiation therapy, and stereotactic radiosurgery (SRS) are common therapeutic approaches to treat cranial metastases. With the improvement in high resolution imaging and patient treatment localization, SRS has a larger role in the treatment of multiple intracranial metastases^1^. SRS is most commonly delivered using a Gamma Knife unit or Linac based system^2^. Traditionally, Linac based SRS for multiple brain metastases utilizes a multiple isocenter approach, aligning each isocenter to the individual metastatic lesion and treating each lesion individually. This results in increased treatment time, where the treatment time increases proportionally to the number of lesions treated, due to patient re-positioning for each isocenter and the increased number of treatment beams.

Volumetric modulated arc therapy (VMAT) is a method of intensity modulated radiotherapy that can achieve conformal dose distributions by simultaneously varying the speed of the gantry rotation, the dose rate of the Linac, and the aperture shape of the multileaf collimator (MLC)^3^. Recently there has been significant interest in evaluating the viability of single isocenter Linac based VMAT for the treatment of multiple brain metastases^4^,^5^,^6^. Several studies have been performed comparing single isocenter VMAT to more traditional methods of treatment such as intensity modulated radiotherapy (IMRT), dynamic conformal arc (DCA), and 3D conformal radiotherapy^7^,^8^,^9^,^10^. These studies found that the plan quality of the single isocenter VMAT was equivalent or better than the other treatment techniques, and the treatment time decreased significantly. However, the low dose volume to normal brain increased significantly, up to 46% in some cases^8^. These studies led the authors to conclude that single isocenter VMAT approach has significant high delivery efficiency without sacrificing plan quality, but with the disadvantage of increased low dose spillage to normal brain tissue. Studies have shown that the volume of normal brain tissue receiving low doses of radiation correlates with development of radiation necrosis after SRS^11^,^12^,^13^,^14^. Thus, the single isocenter VMAT plan quality needs further improvement by reducing the low dose spillage.

The increase in low dose spillage for the single isocenter VMAT plans has been proposed to be the result of the larger jaw openings allowing for increased leakage dose between the leaves^9^,^10^. A larger effect may be the result of the island blocking problem, as proposed by Kang J *et al*.^15^. The island blocking problem occurs when two or more targets share the same MLC leaf pair, resulting in an area of normal brain tissue that is not blocked by the MLCs as shown in Fig. 1. The purpose of this work was to develop an efficient optimization algorithm to reduce dose to normal brain tissue by optimizing treatment geometry in radiosurgery of multiple metastases with single isocenter VMAT.

## Methods and Materials

We have developed a projection summing optimization algorithm to solve the island blocking problem. The algorithm includes (a) measuring coordinates of outer boundary points of each lesion using the Eclipse Scripting Application Programming Interface (ESAPI, Varian Medical Systems, Palo Alto, CA), (b) projecting the outer boundary points of the lesion on the BEV plane, (c) calculating the optimal couch and collimator angles with the least total open beam area on the BEV plane for each arc, and (d) generating treatment plans using the optimized angles.

### Optimization of Treatment Geometry

#### Measurement of the coordinates of outer boundary points of lesions

**Figure 2:**
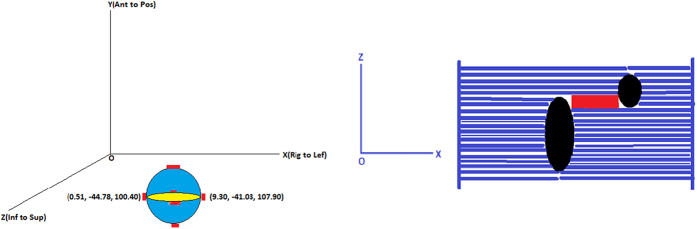
Schematic illustrations of measured coordinate values in mm of two outer boundary points of a lesion in blue in the X direction(left), and the projection of two lesions from the BEV (right). The two black ellipsoids are the two lesions, the blue lines represent the MLC leafs, and the red rectangle represents the area unblocked by the MLCs calculated in the new coordinate R (new).

To localize the lesions in the Beams Eye View (BEV), the coordinates of the boundary points of each lesion are measured using the ESAPI. The ESAPI is a programming interface and software library that allows software developers to write scripts to access the treatment planning information in Eclipse. The principle in measuring the outer boundary point coordinates is to find out the minimum and maximum values of the lesion location at each of the 3 orthogonal directions where the MLC leaves intersects transversally with lesion contours at each control point of an arc. Figure 2 demonstrates the six outer boundary points of a lesion (in red). The coordinate system is identified with the DICOM coordinate system, where the X-axis is the right-left axis, the Y-axis is the anterior-posterior axis, and the Z-axis is the inferior-superior axis.

#### Rotations of couch/table, collimator, and gantry around the isocenter

The spatial rotations of the couch, collimator, and gantry are mathematically expressed as three matrices, respectively:


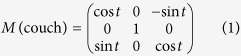



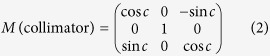



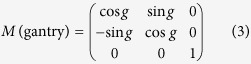


where t, c, and g represent the couch, collimator, and gantry angles, respectively.

#### Projection of outer boundary point of lesion on to BEV projection plane

The rotations of the couch, collimator, and gantry are equivalent to rotations of the lesion(s). The new coordinates, **R** (new), of the lesions after the rotation are obtained from the old coordinates, **R** (old), which were obtained from the ESAPI:


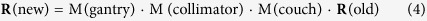


The order of product of three matrices, in this situation should be noted, since M (gantry) does not commute with M (couch) or M (collimator). The projected outer boundary coordinates in the XOZ plane was used to compute the opening space area in the BEV projection plane at any given gantry angle.

#### Optimizing couch and collimator angles

For a given gantry angle, the projection of lesions on the BEV plane is schematically illustrated in Fig. 2. The red rectangle represents the opening space as calculated in the new coordinate R (new). The total area unblocked by the MLCs is calculated by iteratively summing the unblocked area at each control point, approximately 2-degrees apart. The calculations are carried out for all feasible combinations of couch and collimator angles. The couch and collimator angles with the least total unblocked area are then selected as the optimized angles for that specific treatment arc. If the optimization came with multiple combinations of optimal couch and collimator angles, we defined the following selection criteria: (a) keeping treatment arcs equivalently spaced or symmetrical in order to reduce the low dose spread and the gradient index; (b) avoiding the collision between the couch and gantry.

The feasible angles of the collimator ranged from 0- to 165-degrees, due to the limitation of the collimator rotation on the machine. Since the leaves are symmetric, angles 165–360 were not included. The range of the couch angles was determined in order to avoid collisions between the couch and gantry. The possible range of couch angles was selected as follows:


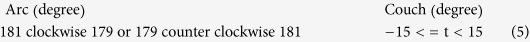



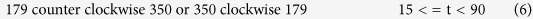



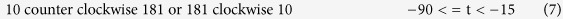
3 to 4 partial or full arcs were used in the treatment plans. The corresponding couch and collimator angles were optimized with 5-degree intervals for each arc. Plans generated with the optimized couch and collimator angles were compared to plans generated with the same couch angles and a fixed collimator angle of 45 degree used by Wolff^5^, Kang^15^, and Audet^16^ in order to evaluate the reduction in low dose spillage to normal brain tissue with optimized angles.

### Patient and Plan Characteristics

A retrospective study of thirteen patients previously treated with 3–5 brain lesions was conducted as part of a protocol approved by the Henry Ford Hospital Institutional Review Board. Table 1 summarizes the number and volume of the lesions for each patient. The average volume of the planning target volume (PTV) was 1.00 cc (range 0.02–6.88 cc). The PTV of all the lesions was combined into a PTV_total_ for plan evaluation. The PTV_total_ for each patient ranged from 0.47 to 11.37 cc.

Plans were generated in the Eclipse treatment planning system. The progressive resolution optimizer (V13.5.35) was used for VMAT optimization and the anisotropic analytical algorithm (AAA, V13.5.35) with a grid size of 1mm was used for final dose calculation. A 6MV high intensity flattening filter free (FFF) photon beam with a maximum dose rate of 1400 MU/min was utilized. Plans were optimized following the single isocenter VMAT planning guideline published by Clark *et al*.^6^. All plans were normalized so that greater than 99% of the PTV_total_ received the prescription dose of 18 Gy.

The isocenter was placed at the geometric center of the PTV_total_. The parameters used to evaluate the dose to the normal brain tissue included the volume of normal brain that received 4, 6, 8, 10, and 12 Gy (V4, V6, V8, V10, and V12, respectively), as well as the mean dose to the normal brain tissue. Plan quality amongst the plans were evaluated using the conformity index (CI), defined as the ratio of the volume of the prescription dose over the volume of each individual PTV; the homogeneity index (HI), defined as the ratio of the maximum dose over the prescription dose; and the gradient index (GI), defined as the ratio of the volume of 50% of the prescription dose over the volume of 100% of the prescription.

## Results

**Figure 3:**
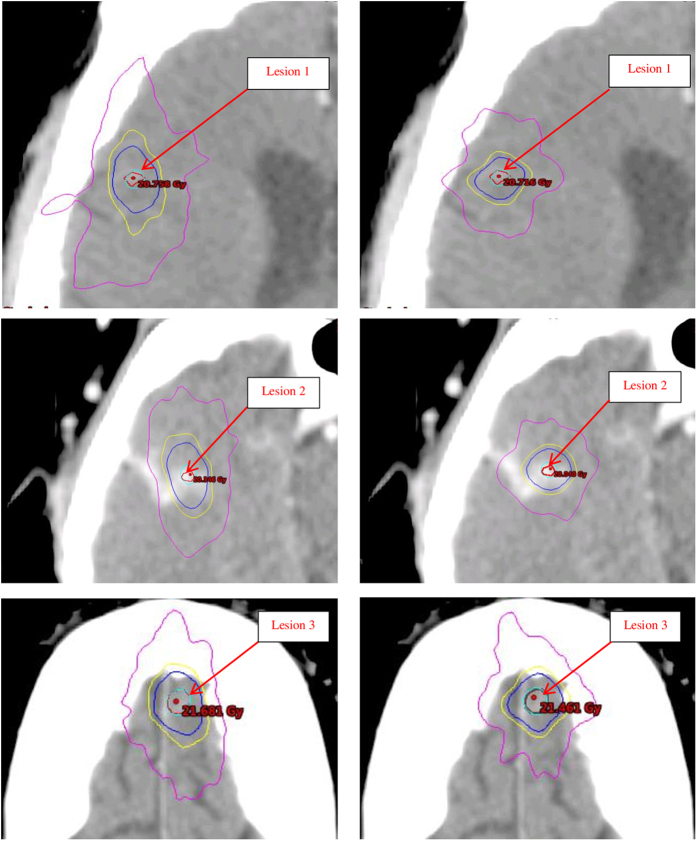
Isodose distributions of two plans for patient 1 with 3 lesions: Left-without optimizing angles of couch and collimator; Right-with optimizing angles of couch and collimator. Dose levels: Purple-4 Gy, Yellow-8 Gy, Blue-10 Gy, Cyan-18 Gy.

**Figure 4:**
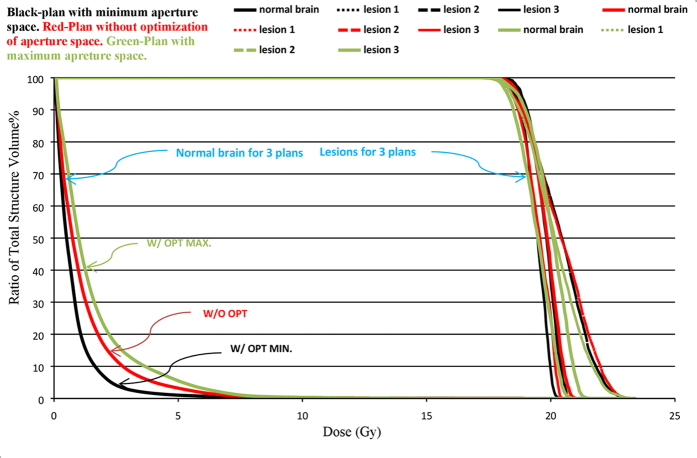
DVHs of the 3 plans. Two with optimized angles selected from minimum beam aperture space (Black - W/OPT MIN) and selected from maximum beam aperture space (green - W/OPT MAX). The third plan without optimized angles (red - W/O OPT). Similar coverage of each lesion is also illustrated within the three plans.

Thirteen patients with 3 to 5 brain metastatic lesions were studied in this work. The optimized couch and collimator angles of each arc were obtained from the Projection Summing Optimizing Algorithm and used to generate treatment plans for each patient. For instance, Table 2 lists the values of optimized angles of couch and collimator for patient 1 with three lesions. According to the selection criteria described previously, these couch and collimator angles were chosen for each of the three arcs: (0°, 10°); (45°, 5°) and (315°, 5°). Figure 3 shows the comparison of dose distributions of two plans with and without optimized couch and collimator angles for this patient. Better dose conformity was achieved in the plan with optimized couch and collimator angles. Quantitatively, 69%, 60%, 32%, 26%, 21%, and 35% reductions were obtained respectively in the V4, V6, V8, V10, V12, and mean dose of normal brain using optimized angles. Figure 4 displays the DVH curves of the lesions and normal brain of the three plans: without optimization, and optimized for the least and most open space area unblocked by the MLCs. The optimized plan with the maximum opening space area was generated to demonstrate the worst case scenario.

Table 3 shows the percent reduction of dose volume, mean dose of normal brain, and mean dosimetric indices CI, HI, and GI of all lesions with and without optimal couch and collimator angles. The mean ± standard error of the percent reduction of the V4, V6, V8, V10, V12 and mean dose of the normal brain was 17.6 ± 5.1 (p = 0.0003), 13.7 ± 4.7 (p = 0.0005), 6.8 ± 2.5 (p = 0.003), 4.2 ± 2.1 (p = 0.004), 3.1 ± 1.6 (p = 0.00005), and 8.4 ± 2.9 (p = 0.006), respectively if optimal collimator and couch angles were used in the treatment plans. The dose volume of normal brain tissue and mean dose of normal brain were reduced significantly due to using the optimal couch and collimator angles. The mean ± standard error of the CI, HI and GI were 1.24 ± 0.04/1.25 ± 0.04 (p = 0.245), 1.16 ± 0.02/1.16 ± 0.02 (p = 0.111), and 5.24 ± 0.52/5.26 ± 0.51 (p = 0.668) with and without optimization. They were no significant changes to the plan quality after optimizing couch and collimator angles. The plan quality was maintained after the optimization with slight improvement, indeed.

**Figure 5:**
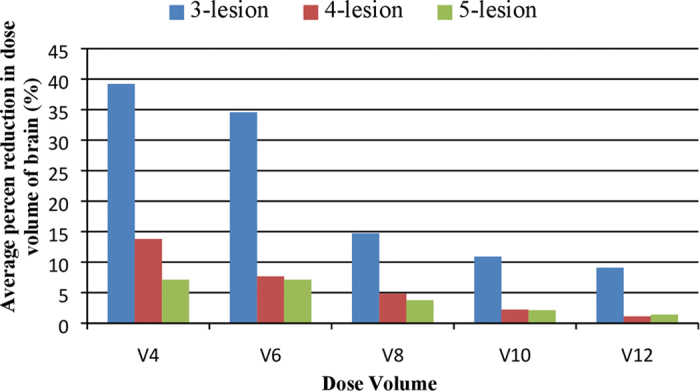
The average percent reduction of the V4, V6, V8, V10 and V12 of normal brain in the three plans with three lesions, six plans with four lesions and four plans with five lesions.

We also evaluated the dependence of the reduction of dose volume of normal brain on the number of lesions. Figure 5 illustrates the percent reduction of dose volumes by averaging all the plans with the same number of lesions. Significant dose volume reduction was achieved on the three plans with three lesions. The benefit of optimization was diminished as the number of lesions increased.

## Discussion

Gamma Knife has been the predominant modality used for confocal treatment of multiple metastases. Linac based SRS treatment becomes attractive because its sub-millimeter targeting accuracy^17^, high delivery efficiency^18^ and excellent plan quality^6^. Previous work has found Gamma Knife to be superior to conformal arc-based multiple target SRS with regard to normal brain exposure^19^, the new generation of Linacs are equipped with a high definition MLC, flattening filter free photon beams (sharper penumbra), jaw tracking and VMAT delivery (less low dose spill to normal brain). Evan M.T. *et al*.^1^ compared the plan quality and delivery time between VMAT and Gamma Knife radiosurgery for multiple cranial metastases. Compared to Gamma knife, VMAT produced clinically equivalent conformity, dose falloff, low isodose spill, and reduced treatment time for multiple lesion treatment. Daniel M *et al*.^20^ compared radiation dose spillage from the Gamma Knife Perfexion with that from VMAT during treatment of multiple brain metastases. They found that VMAT plans had more dose to the normal brain tissues than plans for Gamma Knife^20^. However, wider-width MLC was used in their study, which could be a limiting factor to produce high quality plans for small lesions. Instead, in Evan’s study^1^, a high definition MLC with 2.5 mm leaf width was used and led to better plan conformality and lower dose spillage. Single isocenter VMAT to treat multiple targets has a higher efficiency, but the island blocking problem could lead to an increased low dose spillage to normal brain. This island blocking problem could be solved by optimizing the treatment geometry. The pioneer work was done by Kang *et al*. to optimize the treatment geometry in single-isocenter VMAT^15^. They computed the overlapping sonogram of two lesions to seek the optimized angles of couch and collimator. Our Projection Summing Optimization Algorithm calculated the unblocked area directly by projecting the outer boundary points of the lesions in the BEV projection planes. The algorithm has the flexibility with regard to the beam arrangements and can be integrated in the treatment planning system for clinical application directly. Compared to plans using the conventional treatment geometry, the plans generated with optimized couch and collimator angles showed significant reduction in the mean dose and low dose volume to normal brain. The plan quality using VMAT approach can be further enhanced by implementing the Projection Summing Optimization Algorithm.

The reduction of normal brain dose depends on many factors such as the number of lesions, the size of each lesion, and the relative location and amount of overlap of lesions in the BEVs for the specific patient. Our study focused on the optimization of single isocenter VMAT plans for patients with three to five brain metastases considering multiple randomized controlled trials have been carried out using SRS for patients with one to four brain metastases^21^. Patients with more than five lesions have been treated with the whole-brain radiation therapy in our institute to cover other undetected metastases and protect against intracranial relapse. As claimed by Jairam V *et al*., the current evidence of the efficacy of SRS-only treatment with more than four brain metastases was still sparse and limited to retrospective study^21^. We found that in average, the dose volume reduction to normal brain decreased as the number of lesions increased after the optimization of treatment geometry. Even though the island blocking problem could become more problematic as the number of lesions increases, the benefit of dose volume reduction on the normal brain was reduced by optimizing the couch and collimator angles. The tumor volumes studied in this work were relatively small. The reason is that our SRS program typically treats patients with intracranial lesions that are small or that have a minimal mass effect. The local control of larger tumors with SRS could be compromised since the prescription dose has to be limited to reduce integral dose to the normal brain. The blocking space would increase as the size of tumor increased. The optimization could minimize the blocking spaces effectively and reduce the normal brain dose correspondingly. A study on a larger cohort of patients is still needed to further investigate the correlation of multiple factors on the dose reduction in normal brain.

This study was limited by not showing follow-up MR imaging to demonstrate the effectiveness of reducing radionecrosis using the algorithm. In order to evaluate the performance and safety of the algorithm before applying it to the patient care directly, we applied the optimization algorithm to the patients treated previously and compared the plan quality with and without the optimization. However, it has been demonstrated in many studies that dose volume data during SRS correlates with the development of postradiosurgical imaging changes suggestive of radiation necrosis. The Karolinska group developed a model to predict the risk for radiation induced complications following Gamma Knife radiosurgery for 1128 AVM patients^22^. They concluded that the risk of complications was dependent on the dose distribution, clinical history, AVM location. Flickinger *et al*. at the University of Pittsburgh studied the toxicity of 307 AVM patients treated with Gamma knife with post treatment MRI follow-up. 30% of patients had postradiosurgical imaging changes^23^. Multivariate analysis showed that symptomatic postradiosurgical imaging changes correlated significantly with12 Gy volume^23^. However, most of these studies were published decades ago and focused on the AVM patients treated with Gamma knife radiosurgery. Voges *et al*. correlated the risk of symptomatic radiation necrosis to 10 Gy volume for AVM patients treated with Linac based SRS^24^. In a recent study at the University of Cincinnati, a single institution retrospective analysis was performed for a total of 125 patients with 345 intracranial metastases received Linac based SRS treatment^12^. Radionecrosis was diagnosed based on follow-up MR imaging and histologic evidence. Multivariate regression analysis showed that 8 through 12 Gy volume were the most predictive factors for both symptomatic and asymptomatic radionecrosis in Linac based single fraction SRS of brain metastases^12^. Other factors such as plan conformality and dose inhomogeneity have also been investigated in many studies too. Neither conformality nor dose inhomogeneity were found to correlate with post radiosurgical symptomatic imaging changes^12^,^13^,^23^,^25^. Therefore, we think dose volume data of normal brain in the SRS is probably one of the best predictors of radionecrosis. We have demonstrated that by implementing our optimization algorithm, the dose to the normal brain could be reduced effectively. It would significantly reduce the risk of radionecrosis. Our next step is to enroll patients in a clinical trial with follow-up MR imaging to demonstrate the clinical benefits of the optimization algorithm.

## Conclusions

A projection Summing Optimization Algorithm was developed to address the island blocking problem and has showed great improvement in reducing the low dose to normal brain tissue, while the similar dose coverage to the lesions was maintained. The algorithm can be a robust option to planners to select optimal couch and collimator angles for reducing normal brain dose. The results of the present study suggest that we can achieve a significant dosimetric advantage using this algorithm in single-isocenter VMAT to treat patients with multiple brain metastases.

## Additional Information

**How to cite this article**: Wu, Q. *et al*. Optimization of Treatment Geometry to Reduce Normal Brain Dose in Radiosurgery of Multiple Brain Metastases with Single-Isocenter Volumetric Modulated Arc Therapy. *Sci. Rep.* **6**, 34511; doi: 10.1038/srep34511 (2016).

## Figures and Tables

**Figure 1:**
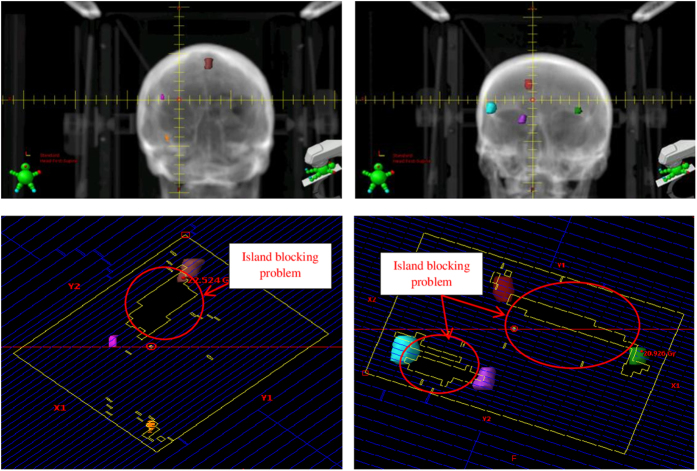
Anterior and posterior DRRs (upper) and MLC leaf pairs shared by two lesions (bottom) resulting in island blocking problems in the BEV projection planes indicated in red for a 3-lesion patient (left) and a 4-lesion patient (right).

**Table 1:** Patient characteristics.

Patient number	No. of lesions	Volume of each lesion (cc)					PTV_total_ (cc)
1	3	0.46	0.04	0.02			0.52
2	3	0.12	0.16	0.19			0.47
3	4	0.33	0.51	0.36	1.16		2.36
4	4	0.33	0.38	0.68	0.12		1.51
5	4	2.63	1.57	1.83	0.76		6.79
6	4	0.15	0.17	0.75	0.42		1.49
7	5	0.31	0.25	0.42	0.75	0.29	2.02
8	5	0.23	1.16	0.25	2.73	0.22	4.59
9	5	0.31	0.09	0.39	1.27	0.50	2.56
10	5	0.84	1.81	1.42	1.06	0.77	5.90
11	3	1.15	2.21	1.48			4.84
12	4	4.05	4.06	0.11	0.39		8.61
13	4	6.88	2.24	1.65	0.60		11.37

**Table 2:** Optimized couch and collimator angles for each arc of patient 1 (degree).

Arc1: Gantry: 181–179 Clockwise		Arc2: Gantry 179–350 Counter Clockwise		Arc3: Gantry 10–181 Counter Clockwise	
Couch	Collimator	Couch	Collimator	Couch	Collimator
350	15	35	40	275	25
0	10	40	10	280	10
5	20	45	5	295	40
10	40	50	100	315	5
		55	140	325	55

**Table 3:** Percent reduction of dose volume, mean dose of normal brain, and mean dosimetric indices CI, HI, and GI of lesions with/without optimizing angles of couch and collimator.

Patient number	V4	V6	V8	V10	V12	Mean dose	CI	HI	GI
1	69.4	60.6	32.7	26.7	21.3	35.5	1.04/1.09	1.18/1.21	6.42/6.30
2	21.2	21.1	5.5	4.0	5.0	9.6	1.58/1.65	1.11/1.12	3.67/3.47
3	12.5	4.7	3.0	0.9	0.5	1.1	1.12/1.12	1.10/1.10	7.20/7.28
4	21.6	13.5	4.1	2.6	1.3	4.7	1.24/1.25	1.12/1.13	5.22/5.18
5	10.3	6.9	4.8	2.3	1.2	3.2	1.12/1.10	1.12/1.12	2.82/2.77
6	8.4	4.9	2.1	0.5	1.7	1.9	1.27/1.28	1.09/1.09	4.39/4.29
7	9.0	4.5	2.0	1.0	2.0	5.4	1.31/1.34	1.11/1.11	5.98/6.07
8	11.3	14.3	10.5	4.7	1.6	3.6	1.26/1.28	1.11/1.12	6.68/6.56
9	2.9	2.2	1.2	0.7	1.1	4.5	1.23/1.28	1.12/1.12	7.03/7.01
10	5.3	7.5	1.3	2.1	0.9	0.8	1.16/1.17	1.14/1.14	8.02/7.92
11	27.0	22.0	6.1	2.1	1.2	18.1	1.23/1.26	1.27/1.27	4.08/4.02
12	20.1	12.9	10.9	5.9	1.0	15.5	1.37/1.31	1.31/1.32	3.70/4.08
13	10.1	3.1	4.1	1.1	1.0	5.9	1.16/1.12	1.29/1.28	3.20/3.23
